# Supplementary material for: Hybrid untargeted short-read and targeted long-read RNA sequencing facilitates genotype-phenotype associations at single-cell resolution
Source: Genome Biol. 2026 Jun 1;27:296. doi: 10.1186/s13059-026-04116-9 (PMC13617763; doi:10.1186/s13059-026-04116-9)
Supplement: Supplementary file 1 [file 13059_2026_4116_MOESM1_ESM.pdf]

Supplementary material: Hybrid untargeted short-read and targeted long-read RNA sequencing facilitates genotype-phenotype associations at single-cell resolution

Jiayi Wang (王嘉谊)[1,2], Maria Constanza Maldifassi[3,4], Anna Bratus-Neuenschwander[5], Qin Zhang (张琴)[5], Felix Beuschlein[6,7,8], David Penton[9] & Mark D. Robinson[1,2,†]

[1] *Department of Molecular Life Sciences, University of Zurich, Zurich, Switzerland*
[2] *SIB Swiss Institute of Bioinformatics, University of Zurich, Switzerland*
[3] *Instituto de Fisiología, Facultad de Ciencias, Universidad de Valparaíso, Valparaíso, Chile*
[4] *Centro Interdisciplinario de Neurociencia de Valparaíso, Facultad de Ciencias, Universidad de Valparaíso, Valparaíso, Chile*
[5] *Functional Genomics Center Zurich, ETH Zurich and University of Zurich, Zurich, Switzerland*
[6] *Department of Endocrinology, Diabetology and Clinical Nutrition, University Hospital Zurich (USZ), 8091 Zurich and University of Zurich (UZH), 8006 Zurich, Switzerland*
[7] *Medizinische Klinik und Poliklinik IV, Klinikum der Universität, Ludwig-Maximilians-Universität, D-80336 Munich, Germany*
[8] *The LOOP Zurich - Medical Research Center, Zurich, Switzerland*
[9] *Electrophysiology Facility, University of Zurich, Switzerland*
[†] *corresponding authors: mark.robinson@mls.uzh.ch*

Contents

1 Supplementary tables **1**
1.1 Data quality . . . 1
1.2 Strand bias . . . 2

2 Supplementary figures **3**
2.1 Data analysis workflow . . . 3
2.2 Data quality . . . 3
2.3 SR-WTA vs. LR-WTA . . . 5
2.4 LR-WTA vs. LR-Twist . . . 8

1 Supplementary tables

1.1 Data quality

Workflow	CellRanger	isoseq
Primer removal	✓	✓
Cell barcode correction	✓*	✓†
CB whitelist	✓	✓
UMI correction	✓	✗
Deduplication	✓*	✓†
Cell calling	✓*	✓†
Reselect sub-threshold cells	✓	✗

Table S1: Summary of processing steps with CellRanger for SR-WTA and isoseq for LR-WTA/Twist. * after mapping; † before mapping.

**Metric**	**Sample A**	**Sample B**
Number of Reads	256,751,486	228,842,856
Estimated Number of Cells	10,174	5,687
Mean Reads per Cell	25,236	40,240
Median Genes per Cell	1,154	1,966
Valid Barcodes	89.2%	90.4%
Valid UMIs	99.9%	99.9%
Sequencing Saturation	31.1%	41.7%
Q30 Bases in Barcode	96.5%	96.0%
Q30 Bases in RNA Read	95.2%	94.2%
Q30 Bases in UMI	97.4%	97.4%
Reads Mapped to Genome	95.1%	94.9%
Reads Mapped Confidently to Genome	68.2%	67.1%
Reads Mapped Confidently to Intergenic Regions	1.0%	0.8%
Reads Mapped Confidently to Intronic Regions	18.7%	15.0%
Reads Mapped Confidently to Exonic Regions	48.5%	51.3%
Reads Mapped Confidently to Transcriptome	60.6%	60.7%
Reads Mapped Antisense to Gene	4.6%	3.6%
Fraction Reads in Cells	60.4%	66.8%
Total Genes Detected	44,778	40,835
Median UMI Counts per Cell	2,191	4,642

Table S2: CellRanger summary metrics for Sample A and Sample B for SR-WTA.

Type	Sample	LR-WTA	LR-Twist
raw	sampleA	85,182,821	35,472,753
raw	sampleB	78,196,246	28,235,749
deduplicated	sampleA	74,820,537	7,217,104
deduplicated	sampleB	67,218,222	5,538,856
cell_calling	sampleA	26,999,791	3,613,091
cell_calling	sampleB	28,196,044	3,187,328
mapped_genome	sampleA	26,862,528	3,591,677
mapped_genome	sampleB	28,180,994	3,185,666
mapped_transcriptome	sampleA	20,295,857	2,541,682
mapped_transcriptome	sampleB	22,538,341	2,490,549
counts	sampleA	19,638,599	2,541,073
counts	sampleB	21,785,336	2,489,980

Table S3: Number of reads at each processing step in the IsoSeq workflow for LR-WTA/Twist samples, following the standard *isoseq* analysis pipeline.

1.2 Strand bias

CYP11B2 lies on the negative genomic strand. In a strand-specific library such as MAS-Seq, reads from *CYP11B2* transcripts are therefore expected to align predominantly to the reverse strand, and the observed strand asymmetry at this locus is consistent with that orientation. Strand specificity fixes the *global* orientation, but strand-bias tests (Fisher exact test) ask whether REF and ALT share the same +/− distribution among the reads that do occur. Even in stranded data, a small fraction of opposite-strand alignments is expected (e.g., rare antisense, capture effects); if ALT is disproportionately enriched in that minority strand while REF is not, this suggests an assay/mapping artifact rather than biology. To evaluate strand bias at *CYP11B2* (chr8:142,912,613 G>A) from the IGV observations, we formed a 2×2 table of forward/reverse counts for REF and ALT and applied Fisher's exact test. The Fisher strand score (FS) was computed as:

$$\text{FS} = -10 \times \log_{10}(p)$$

where p is the two-sided p-value from Fisher's exact test.
For the LR-WTA dataset, the counts were:

	Forward	Reverse
REF (G)	1	15
ALT (A)	1	5

yielding $p = 0.48$ and $\text{FS} = -10 \times \log_{10}(0.48) = 3.18$.
For the LR-Twist dataset, the counts were:

	Forward	Reverse
REF (G)	8	300
ALT (A)	5	43

yielding $p = 0.02$ and $\text{FS} = -10 \times \log_{10}(0.02) = 16.95$.

2 Supplementary figures

2.1 Data analysis workflow

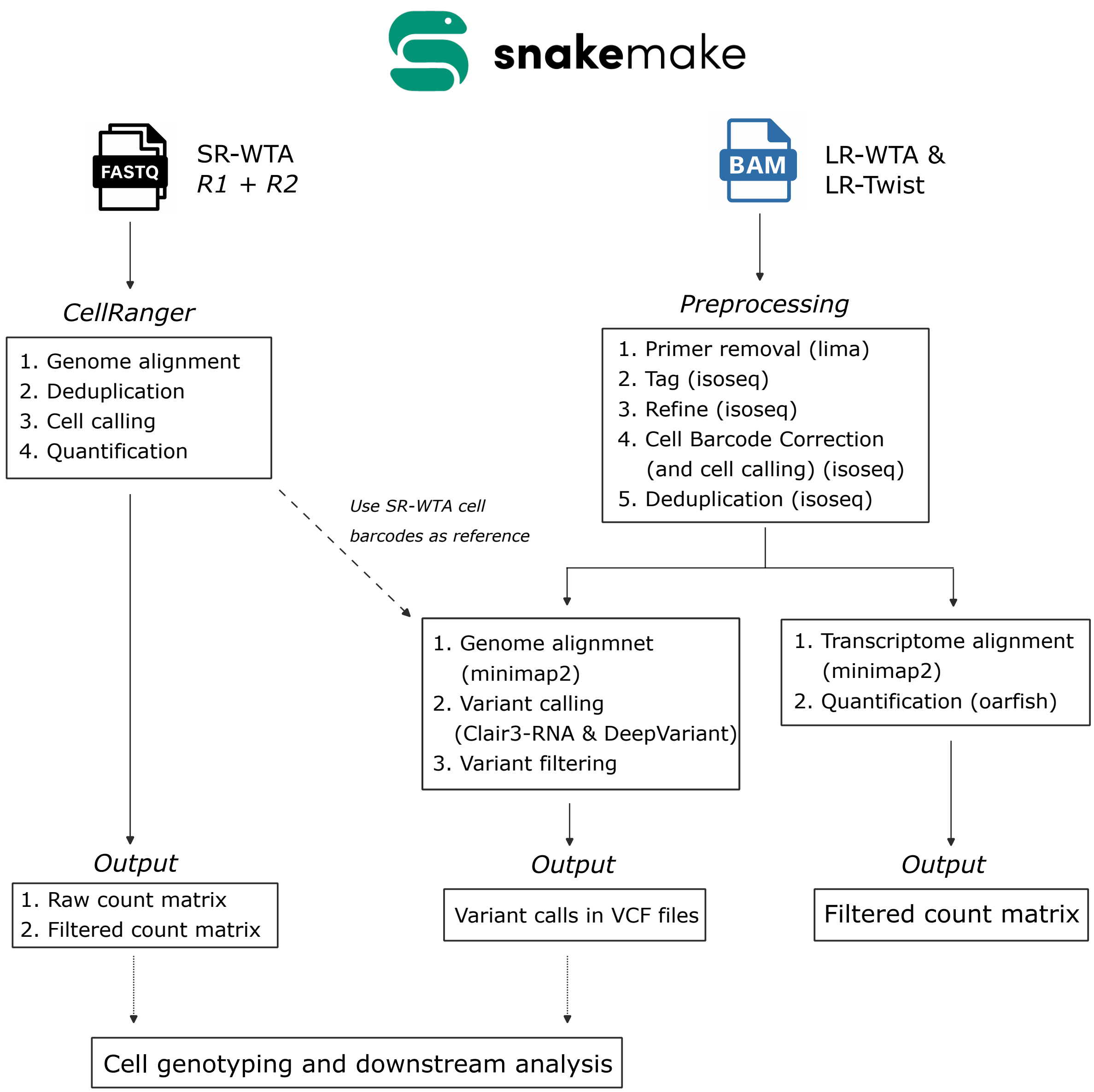

Fig. S1: Schematic Snakemake workflow of data analysis.

2.2 Data quality

We visualized the number of reads retained at each processing step for SR-WTA, LR-WTA, and LR-Twist (Fig. S2). Both *CellRanger* and *isoseq* use a knee-finding method by default to determine the cell barcode threshold. However, for LR-Twist datasets, this approach underestimated the true number of cells because the barcode rank plots displayed a gradual curve rather than a sharp inflection point. To address this, we applied a percentile-based thresholding method using the 95th percentile for the long-read datasets. With this 95% cutoff, the number of detected cells in LR-WTA closely

matched that obtained using the knee-finding method. For SR-WTA, since *CellRanger* does not produce intermediate BAM files, the number of reads at each step was estimated from the metrics reported in Table S2.

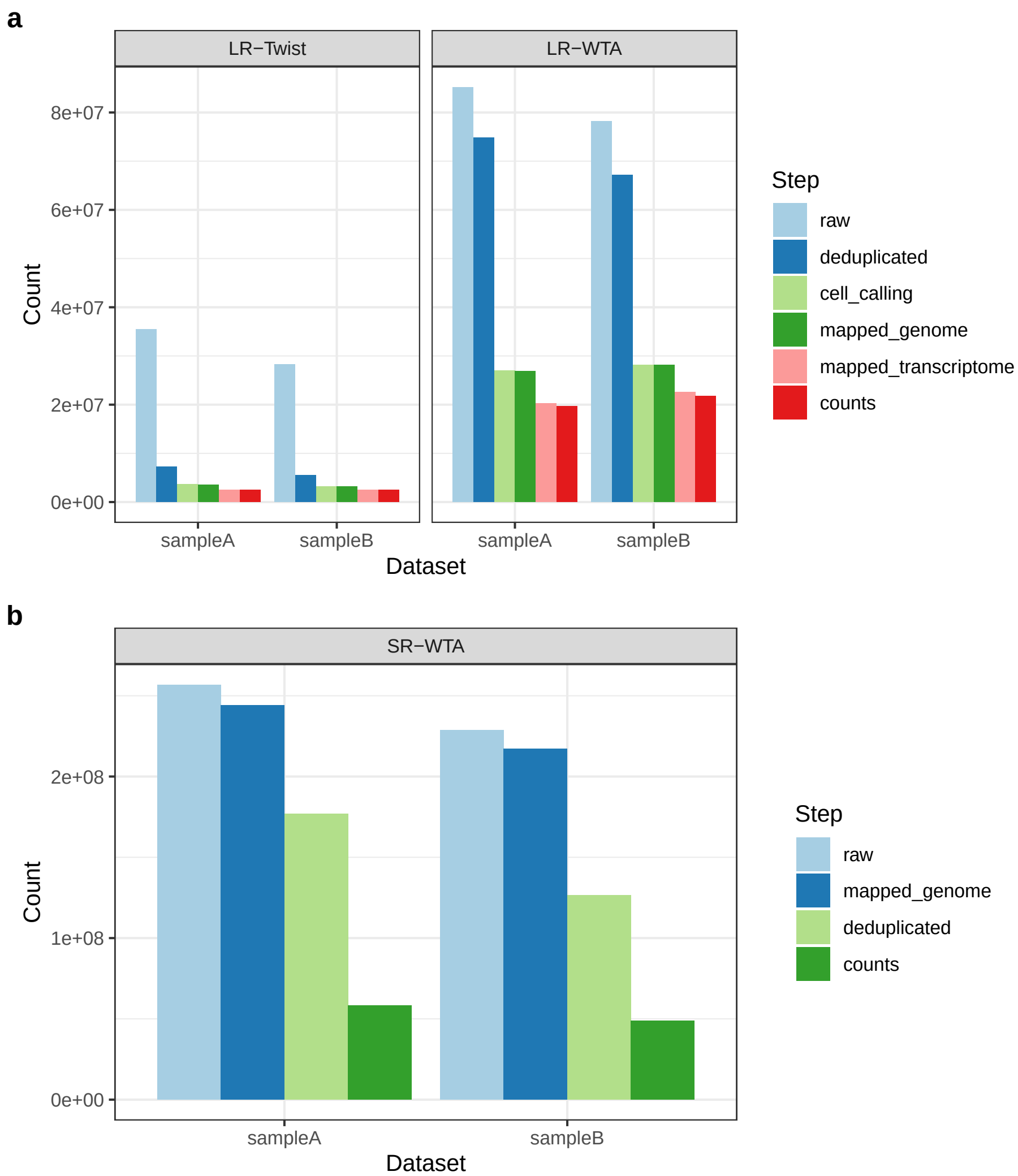

Fig. S2: The number of reads at each data processing step.

2.3 SR-WTA vs. LR-WTA

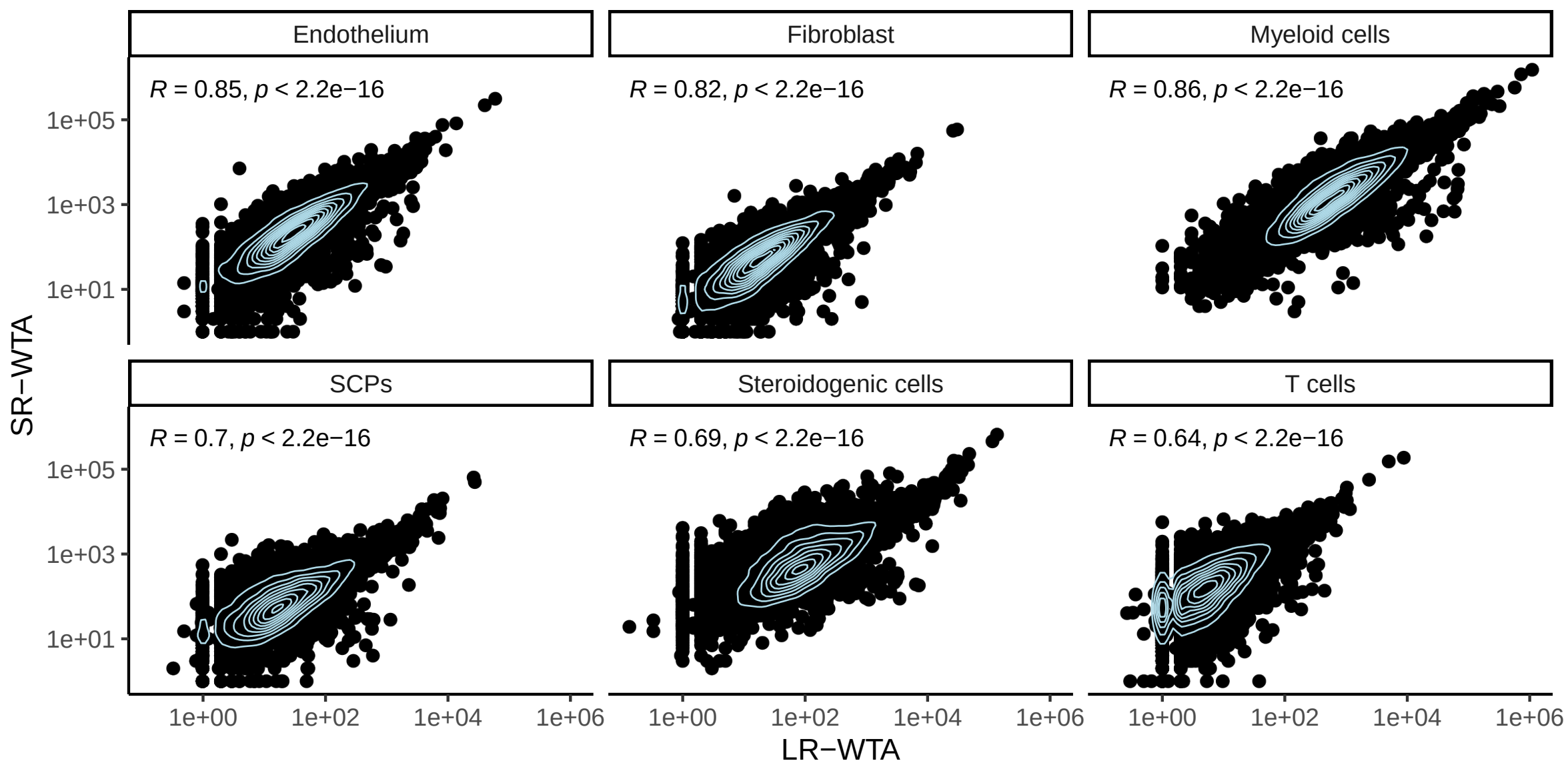

Fig. S3: Spearman's correlation on UMI counts per gene between SR- and LR-WTA, stratified by cell types.

Fig. S4: Comparison of downsampled SR-WTA and LR-WTA using default cell-calling methods. (**a**) Cell barcode rank plots, where the x-axis shows barcodes ranked in descending order and the y-axis represents the total UMI counts per barcode. Points are colored according to whether they were called as cells by the respective default pipelines, i.e., CellRanger for SR-WTA and isoseq for LR-WTA. (**b**) Venn diagrams illustrating the overlap between downsampled SR-WTA and LR-WTA cells, with the first row corresponding to sampleA and the second to sampleB. (**c**) UMAP projections of cells from sampleA and sampleB, colored by cell types annotated using CellTypist. (**d**) Bar plots showing cell type proportions in LR-WTA and downsampled SR-WTA. Panels **c** and **d** share the same color legend.

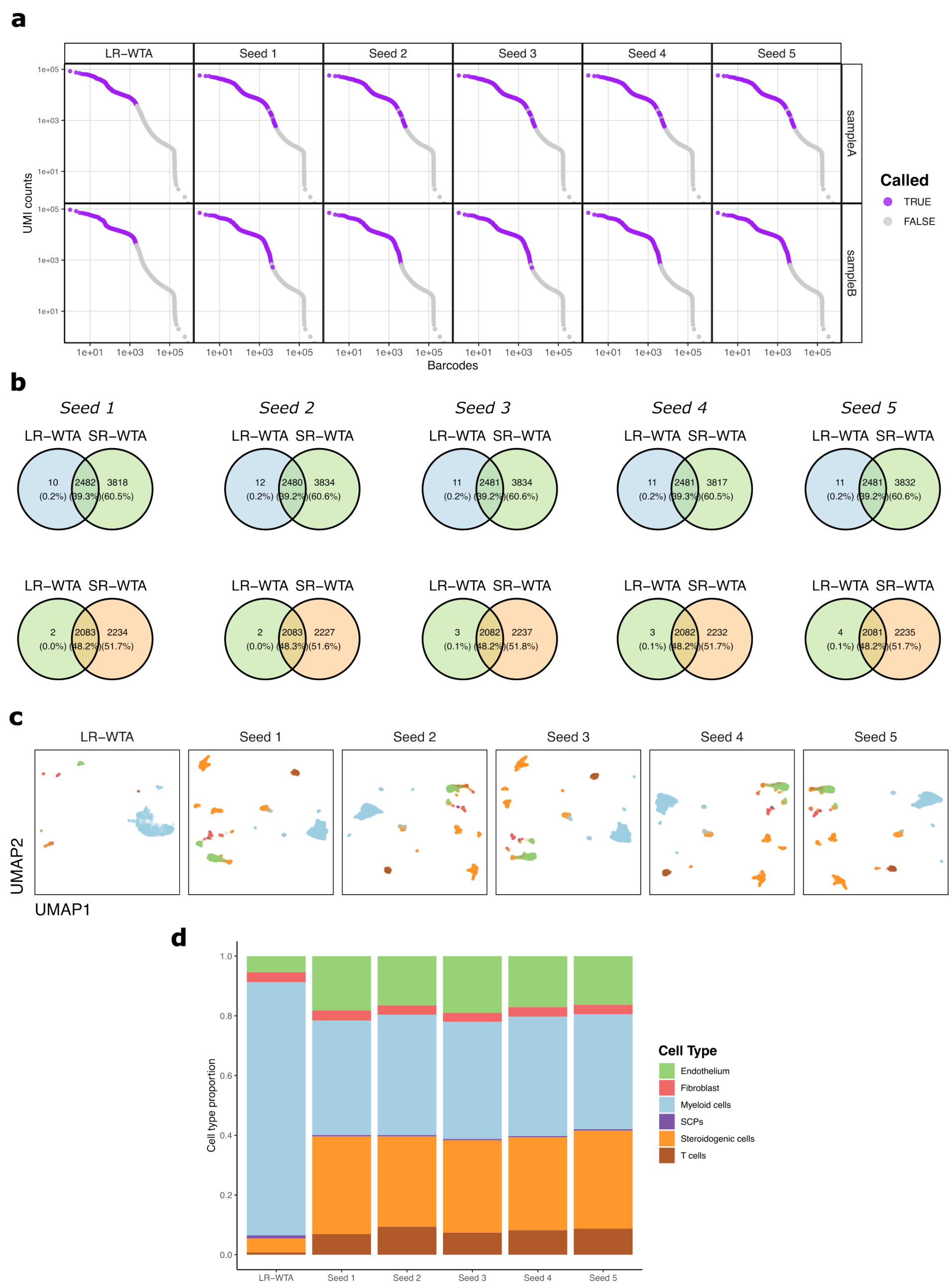

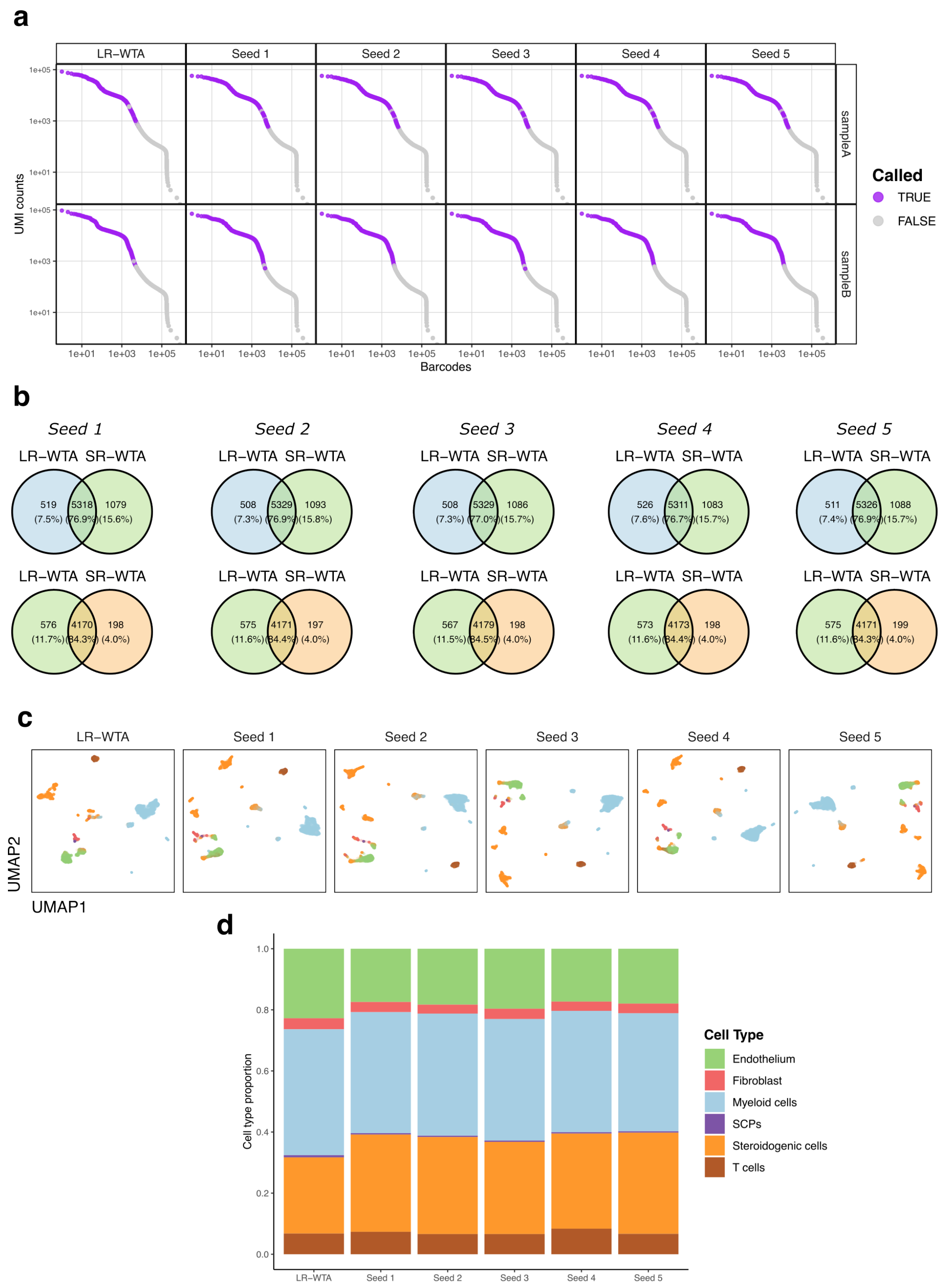

Fig. S5: Comparison of downsampled SR-WTA and LR-WTA using the same cell calling method. Same as Fig.S4, but the same cell-calling method (EmptyDrops) was applied to the raw count matrices.

2.4 LR-WTA vs. LR-Twist

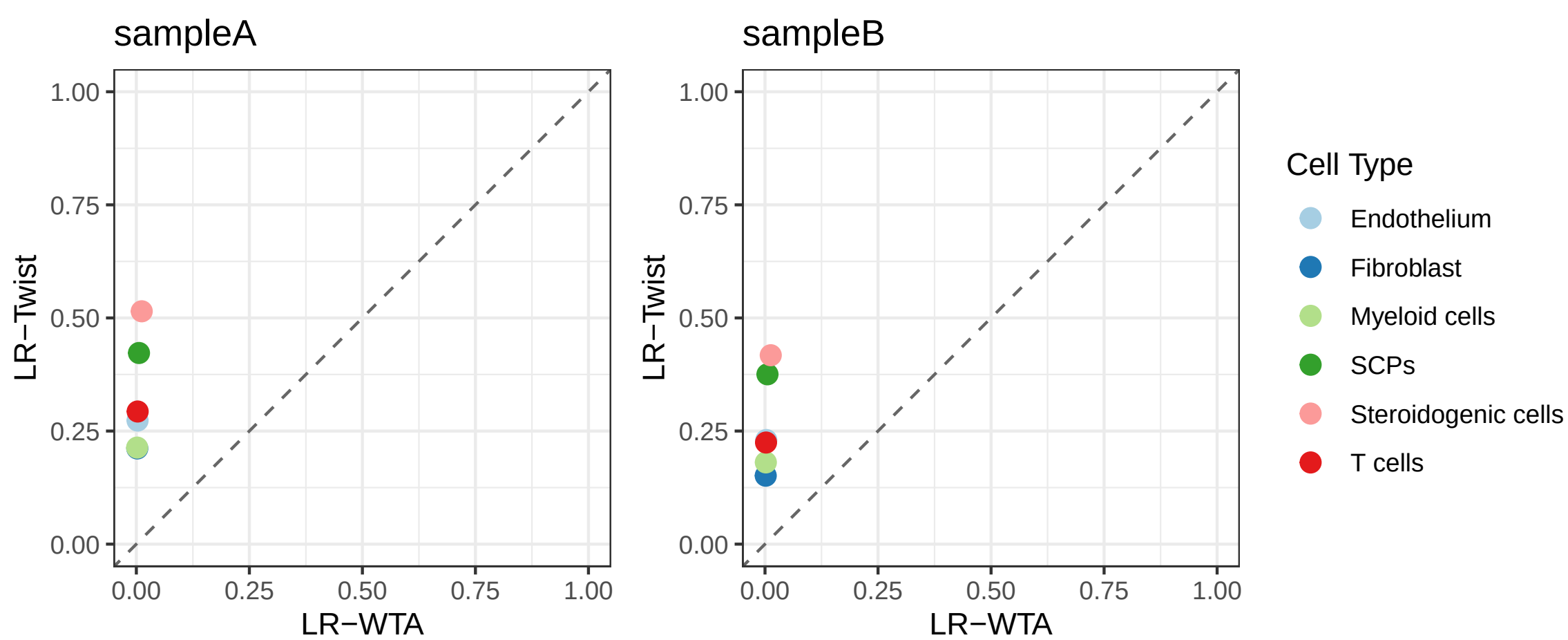

Fig. S6: Proportion of targeted reads in each cell type in LR-WTA and LR-Twist, colored by cell types.

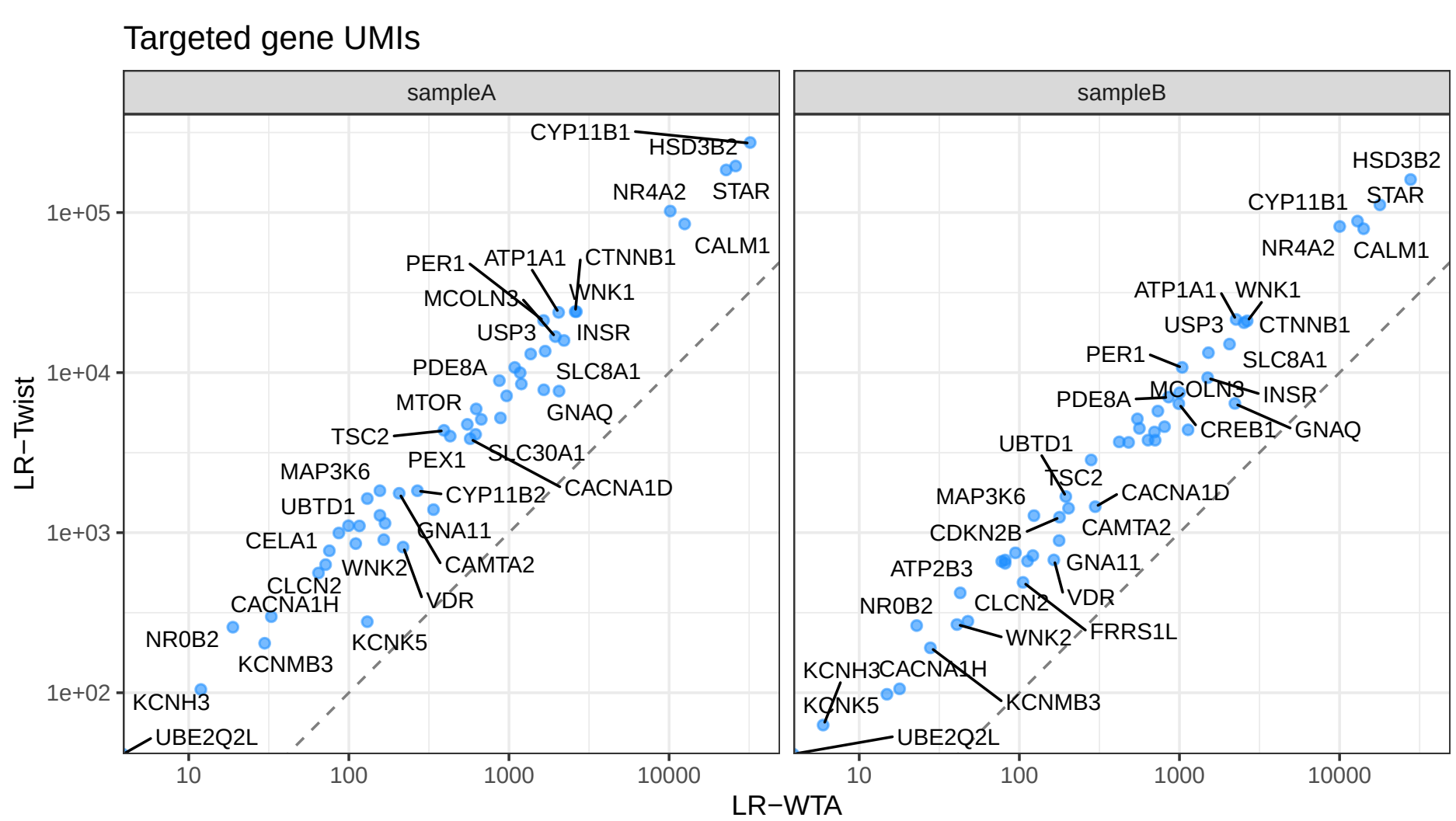

Fig. S7: Twist capture efficiency on targeted genes. Scatter plot comparing summed gene-level UMI counts across all cells between LR-WTA and LR-Twist (on log10 scale), only for targeted genes.

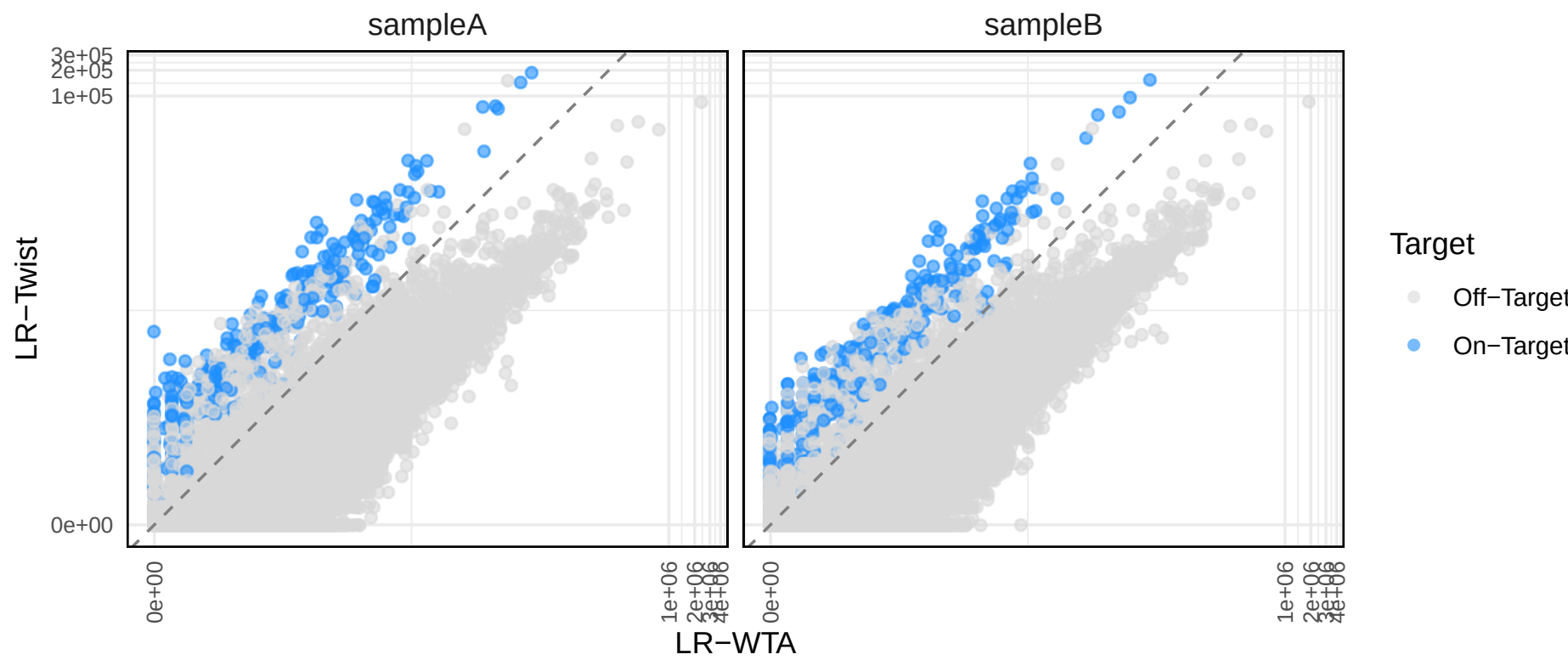

Fig. S8: Twist capture efficiency on targeted transcript. Scatter plot comparing summed transcript-level UMI counts across all cells between LR-WTA and LR-Twist (on log10 scale).

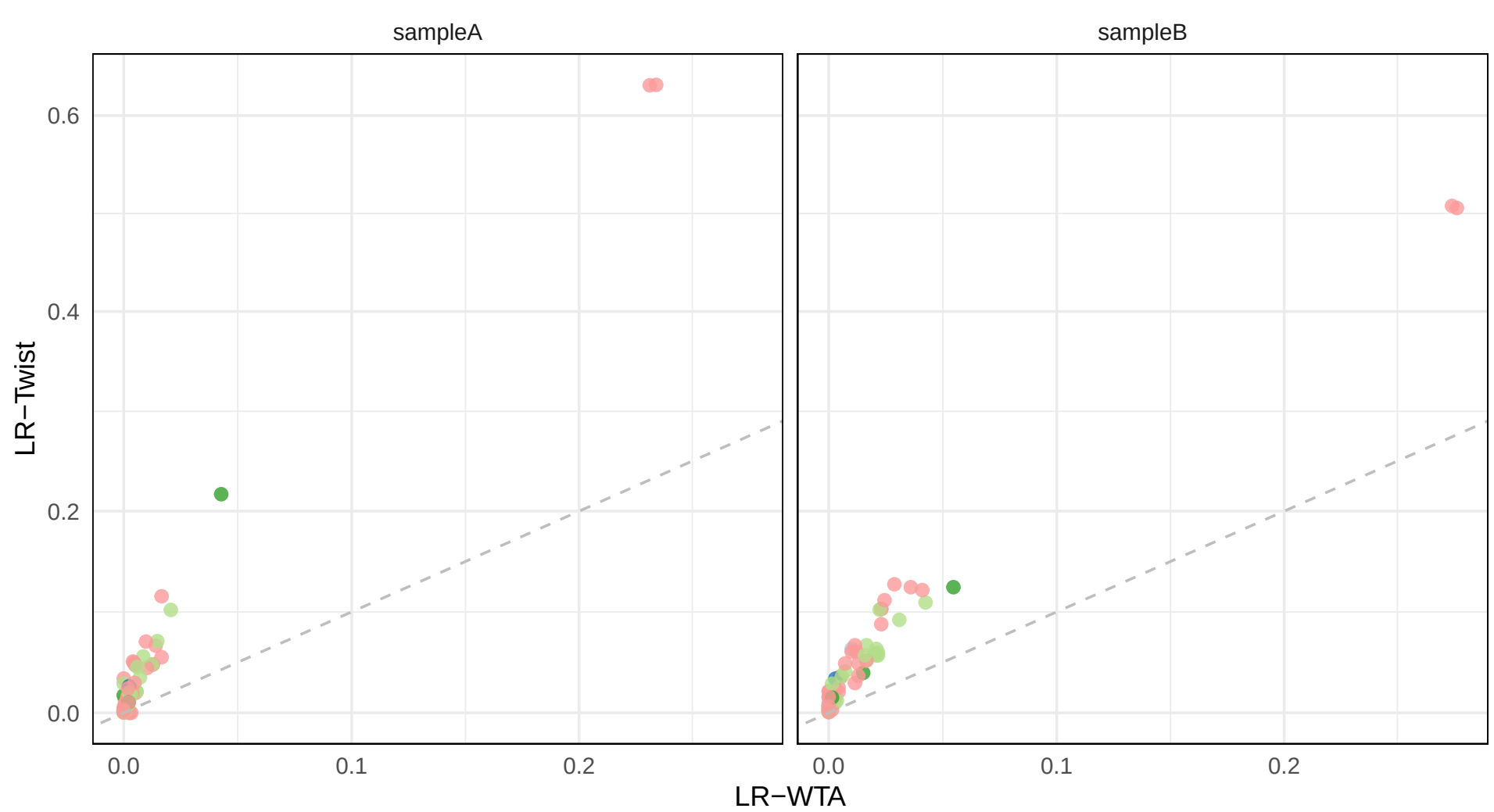

Fig. S9: Proportion of steroidogenic cells genotyped in LR-WTA and LR-Twist. The total number of steroidogenic cells is the same in LR-WTA and LR-Twist, since both are based on the cells identified in SR-WTA.

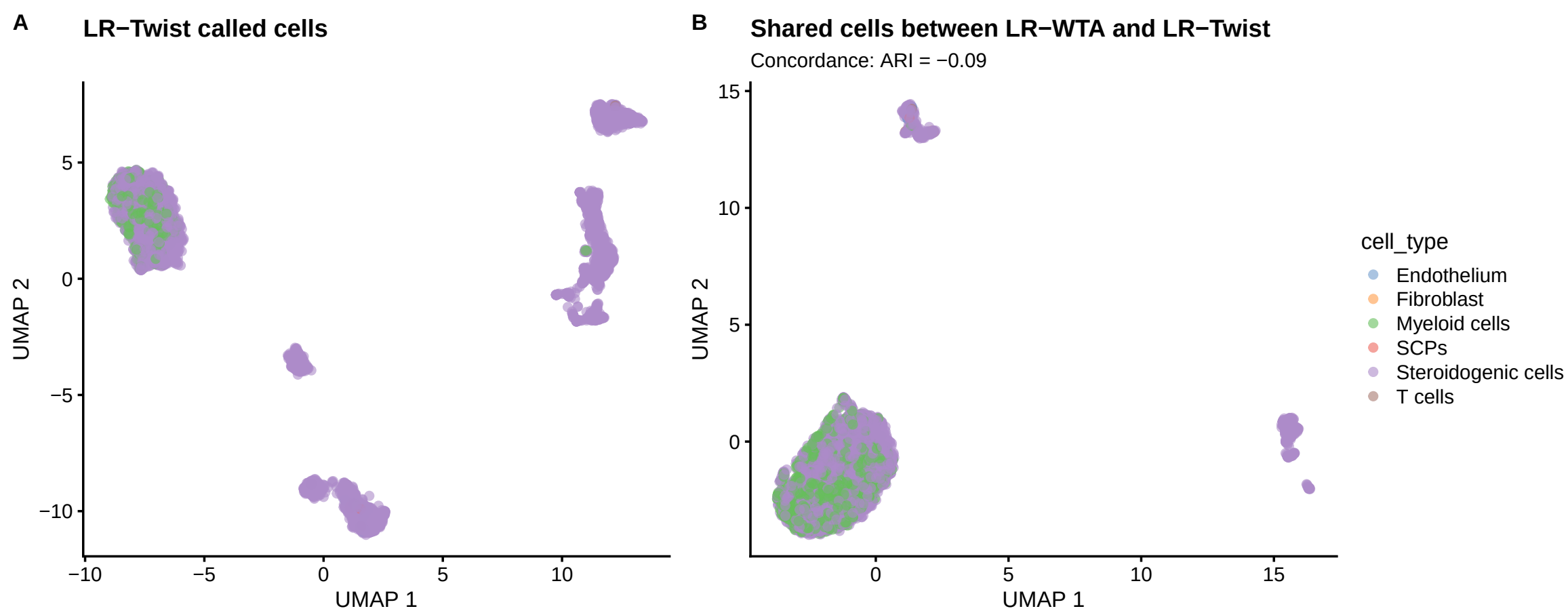

Fig. S10: UMAP projection of cells from samples A and B for LR-Twist. (**a**) UMAP of called cells from LR-Twist. (**b**) UMAP of cells shared between LR-WTA and LR-Twist, annotated with the adjusted Rand index (ARI) computed from the cell type labels of LR-Twist and LR-WTA. Low-dimensional embeddings were generated using the top 3,000 highly variable genes (HVGs), and cells are colored by cell types assigned by `CellTypist`.

Here, we used one gene as an example to illustrate the inefficiency of variant calling from SR-WTA. In Integrative Genomics Viewer (IGV), reads from SR-WTA are concentrated at the 3' end and only a few are mapped to the coding regions, in contrast to LR-WTA and LR-Twist, which show broader coverage across the gene body.

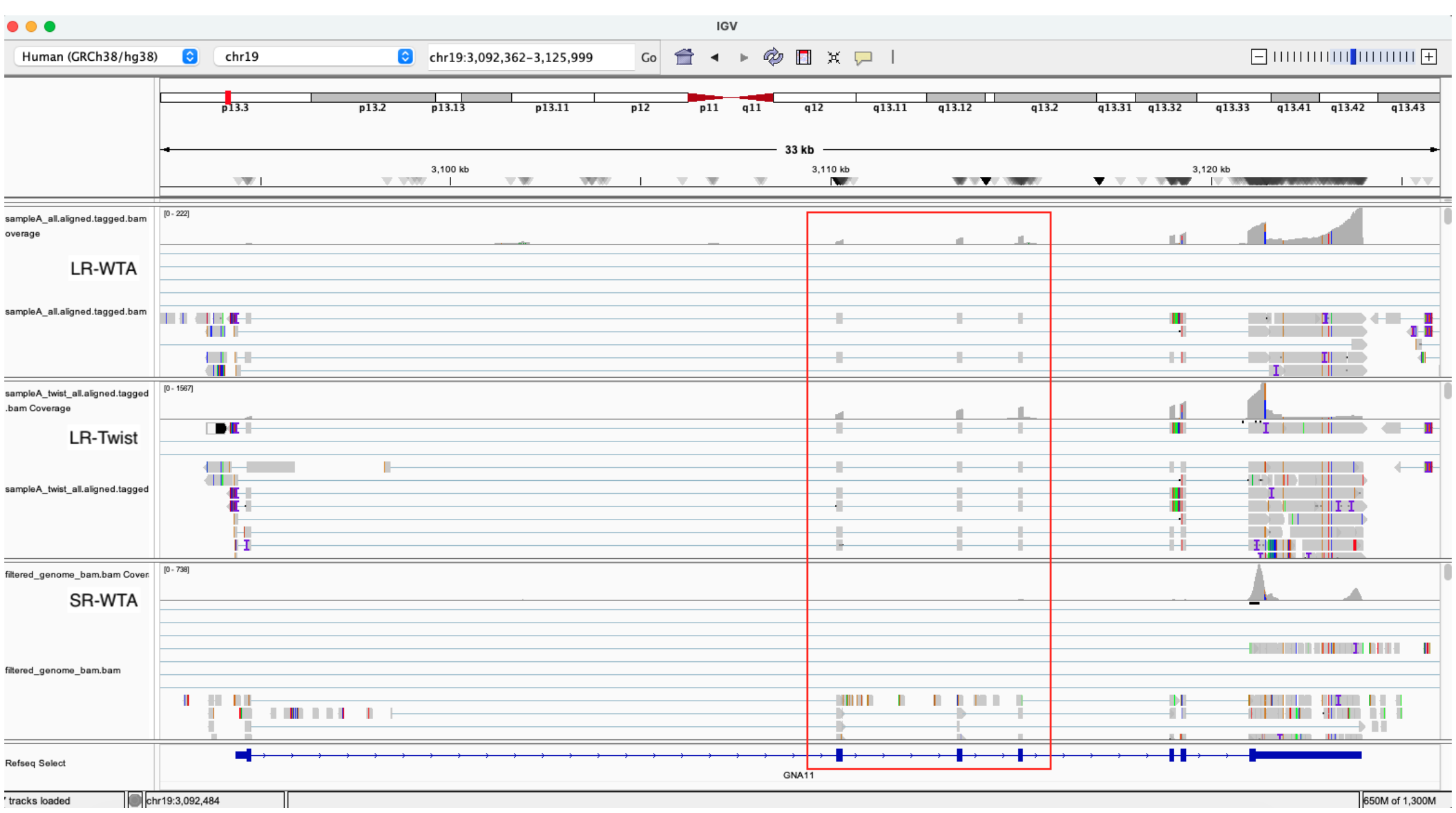

Fig. S11: IGV screenshots of reads mapped to *GNA11* from SR-WTA, LR-WTA and LR-Twist.
